# What makes smartphone games successful in food information communication?

**DOI:** 10.1038/s41538-020-0062-8

**Published:** 2020-01-30

**Authors:** Liran Christine Shan, Julie L. Schiro, Kai Zhong, Patrick Wall

**Affiliations:** 1 Institute of Food and Health, University College Dublin, Belfield, Dublin, 4 Republic of Ireland; 2 Michael Smurfit Graduate Business School, University College Dublin, Carysfort Avenue, Blackrock, Co., Dublin, Republic of Ireland; 3 China Food Information Center, Pomegranate Center, No.88 Liu Xiang Road, Fengtai District, Beijing, People’s Republic of China

**Keywords:** Education, Education

China’s annual Food Safety Publicity Week in June 2019 has, for the first time, included smartphone games in its activities. Food related governmental and non-governmental organizations liaised with Alipay (Alibaba’s payment application) launching a smartphone quiz-game with an unprecedented level of public engagement with food information (i.e., food safety, nutrition, and food science and technologies): 12 million participants and 1.7 billion instances of participation within 1 week. This example demonstrates the great potential of smartphone games, and more widely, digital tools, in food-related public education. Given the extent of misinformation among the public on food safety and nutrition issues, food scientists and organizations should be empowered to embrace emerging tools, such as smartphone games, in order to positively shape public opinions. To aid in this task, this commentary article analyzes the factors behind the unprecedented success of Alipay’s smartphone quiz-game during China’s Food Safety Publicity Week.

## What makes smartphone games successful in food information communication?

Smartphone games present a substantial opportunity to reach and teach the public regarding food information. On June 10th 2019, in collaboration with the State Administration of Market Regulation (SAMR) and the China Food Information Center (CFIC), Alipay—the mobile and online payment application created by Alibaba (world’s largest e-commerce company)^1^—launched a mobile-based quiz-game for the Chinese people: ‘Da Da Xing Qiu (Quiz-game Planet): Food Safety Week’. This quiz-game was part of China Food Safety Publicity Week’s activities. The game challenged users on their knowledge of food safety, nutrition, and food science and technologies. In less than a week, the game rapidly surpassed the benchmarks of previous food safety and nutrition campaigns on reach and engagement^2^ (Table 1).

**Table 1:** Key statistics from Alipay’s Da Da Xingqiu (Quiz-game Planet) Food Safety Week^2,27,28^.

Timeline	Reach and engagement of the smartphone game
Day 1 (10 June, 2019)	• Over 3 million people participated
First 5 days	• 10 million people participated • The participation/click times reached 1.27 billion • 90% of participants shared the food safety knowledge they learned with friends and relatives
First week	• 12 million people participated • The participation/click times reached 1.7 billion

## Secrets behind the success

The unprecedented popularity of Alipay’s quiz-game represents a unique opportunity to study what led to the games’ wide and rapid adoption. The first driver of success involves Alipay’s vigorous promotion of the quiz-game across a range of channels, from digital to traditional. Alipay ran prominently placed banner ads promoting the game on its own websites. Given Alipay’s massive userbase of over 1 billion,^3^ these banner ads were assured massive reach, and reach is a major predictor of advertising success according to longitudinal marketing studies across a range of industries.^4^ Alipay further bolstered this reach by utilizing offline channels, specifically supermarkets. Over 150 Hema Xiansheng fresh food stores (Alibaba-owned online-to-offline supermarkets) and nearly 60 supermarkets under the China Chain-Store & Franchise Association (CCFA) launched promotions to encourage shoppers to participate in Alipay’s game by scanning QR codes throughout the stores.^5^ By involving supermarkets, Alipay could reinforce learnings in a context where the knowledge was highly relevant. And by utilizing both online and offline channels, Alipay increased the likelihood of amplification, whereby online and offline advertising efforts become greater than the sum of their parts.^4^

Alipay’s promotional efforts reached millions of people, but exposure is expensive, and other organizations may have difficulty affording even a fraction of this reach. Indeed, the value of the Alibaba-owned advertising space dedicated to Da Da Xing Qiu is estimated to be around 6.3 million RMB, or about 900,000 USD. While this price tag is unaffordable for most small food safety and nutrition organizations, there is good news. Most large companies (including Alibaba) have corporate social responsibility (CSR) initiatives that support external prosocial initiatives such as the China Food Safety Publicity Week. Indeed, Alibaba covered the cost of the quiz-game development and donated the advertising space in its stores and online. Hence, small food safety and nutrition organizations may be able to capitalize on the CSR initiatives of big companies and influencers to help promote their campaigns, despite modest budgets.

The second driver of success involves ease of access. Typically, smartphone games require players to download, install, and eventually uninstall software. Some games further require users to create an account, enter payment information, and link their social media profiles. This was not the case for Alipay’s quiz-game. Alipay’s quiz-game was released as a “mini-program.” Mini-programs are sub-applications which automatically load as part of a larger application. This meant that users of Alipay’s mobile app could easily access the quiz-game by entering ‘Da Da Xing Qiu’ (Quiz-game planet) in the search panel of the Alipay front page without needing to download or install any software. Ease of access is critical to smartphone success; the fewer the steps required to complete a task on a mobile device, the higher the mobile engagement and conversion rates.^6^ Not only did Alipay reduce the steps required to play the game, they also made it easy to share the results with others. And sharing, especially on social media, is instrumental in propelling a campaign’s reach further.^7^

Mini-programs have the added benefit of being quicker and cheaper to develop than traditional mobile applications since they are built on top of a main application, which means they often fit better with the limited budget of public agencies and small organization.^8,9^ For the end users, mini-programs typically load quicker than stand-alone games and can be easily shared with other users of the main application.^8,9^ A caveat, of course, is that the success of mini-programs requires a substantial userbase of the main application.

A game-based format matters as well. With the advent of digital media, people have numerous options vying for their attention. This makes it difficult for food safety information to stand out. A game can better compete in this cluttered space by being entertaining and offering prizes, even if the goal is ultimately to educate. In the case of Alipay, users earned points by taking the quiz which could be redeemed for monetary credit on Alipay. Scholars have suggested several gamification design tactics that stimulate high levels of engagement with smartphone games: play or entertainment, challenge, real-time feedback, and reward.^10,11^ Alipay’s quiz-game reflected all four (Table 2).

**Table 2:** Tactics implemented in the design of quiz-game.

Tactics	Description
Play or entertainment	Participants could choose to play alone or join a mulit-player session to compete with others. There was also a leader board, which dynamically ranked participants’ achievements against peers.
Challenge	The question pool included 1,000 questions under five themes: ‘Food rumors’, ‘Internet famous food’, ‘Knowledge about tea’, ‘Staying away from junk food’, and ‘Healthy eating for families’. There was a good mix of common-sense questions and difficult ones. The questions were randomly selected at each round of participation. Participants could repeat the quiz-game as many times as they wanted.
Real-time feedback	Participants received feedback after answering each question: they saw a pop-up box briefly explaining the related food safety fact, and the percentage of other players who got the question correct. This allows participants to address their knowledge gaps or misperceptions immediately.
Reward	Each round of participation resulted in an opportunity to win a prize (e.g. points on Alipay, cash, or payment-in-kind, etc). As users accrued points or ‘leveled up’, their chances of winning a prize increased.

The fourth driver of success involves the sheer ubiquity of smartphones, and the popularity of smartphone games in China according to the China Internet Network Information Center and Apple app store.^12,13^ In China, 57.7% of the population are mobile internet users.^12^ Numbers are even higher globally. A median of 75% of adults in advanced economies and 45% in emerging economies own a smartphone.^14^ Numbers are higher still among the youth. For instance, 95% of teenagers and 96% of young adults own or have access to a smartphone in the US.^15,16^ Smartphones are also a major part of daily life. For instance, the average American checks their phone 52 times a day,^17^ with similar numbers observed in Europe.^18^

The last driver of success is that the quiz appealed to multi-taskers, short attention spans, and people on the go. Participants could join the quiz at any time and any place (via a mobile device). They could participate in a few seconds, learn a few facts via the real-time feedback, then quit the game before ever “completing” it. Such design fits the new normal of many mobile phone users’ behaviors: their attention span has become shorter and they are less inclined to immerse themselves in any lengthy text-based educational materials.^19^

Among all aforementioned factors, the most critical was likely Alipay’s vigorous promotion efforts which resulted in mass exposure of the quiz-game to the public. Many mistakenly believe that high-quality content (including attractive smartphone games) requires very little investment in promotion because it will “go viral” organically. That is, one person will share it with two people who will share it with four people and so on, ultimately driving exposure into the millions. But content rarely, if ever, goes viral in this way.^4,20,21^ Indeed, several recent food-related smartphone games have failed to go viral despite being of high-quality. Two examples are “Just Food Fun” (a quiz-game application testing food literacy) and “Are you ready for food preparation 你适合下厨房么” (a mini-program quiz-game examining users’ cooking practices against WHO’s food safety guidance). These games have, to varying extents, demonstrated similar design features to the games created by Alipay, yet have failed to reach similar engagement metrics. A key difference between these quiz-games and Alipay’s quiz-game is the level of initial promotion. Alipay promoted the quiz-game to its billion-level userbase through its online and offline advertising channels. It is this type of mass-market coverage that often triggers buzz and kickstarts sharing, resulting in what we commonly think of as “organically” viral content.^4,20,21^

The power of Alipay’s reach and promotion is difficult to match, and helps explain why Alipay’s other quiz-games have also seen such rapid traction. For example, one of Alipay’s quiz-games on personal finance and financial security was played 200 million times in the first 20 days.^22^ One may argue that most organizations neither have the userbases to emulate the success of Alipay nor the means to afford an investment in promotion and public relations to secure coverage in the millions. However, *partnership* can be a remedy here, either with other organizations, influencers, the media, et cetera, to build a broader reach. As noted earlier in the paper, influential sources or platforms may be willing to donate resources, including promotion space, for a good cause under the umbrella of social responsibilities.

## Additional benefit of smartphone games: data

In addition to the massive scale of public participation, smartphone games also provide quick and real-time insights on the populations’ knowledge levels. Public education campaigns typically start with research to identify problematic knowledge gaps. Traditionally, this has been done through effortful research (e.g., surveys, focus groups) and involves a long time span from data collection to analysis and reporting. But digital tools can greatly streamline and expedite this process in real-time and at a scale that is far beyond the capability of manual analysis. Taking this Food Safety Publicity Week as an example, only 8 days after the mobile quiz-game had been launched, the CFIC publicized insights from the data—records from the first 300 million times of participation.^23^ This was the first ever big-data-driven profiling of Chinese consumers’ food safety and nutrition knowledge. The data showed where public knowledge was weak, giving communicators direction for future food safety communications.

## Further thoughts and suggestions

It remains unclear whether smartphone games are capable of generating strong and long-term educational and behavioral outcomes. In the case of Alipay’s quiz-game during the Food Safety Week, data on the long-term impact were not available. However, given the massive participation in Alipay’s quiz-game, there is no doubt that it had at least some measurable impact on public knowledge.

It is, of course, critical for firms to evaluate the success of their campaigns, often operationalized as increased knowledge or behavioral outcomes. Evaluating these should be planned and integrated from the very beginning of the communication campaign, achieved through multi-stage surveys, including surveys conducted before the campaign (to establish baseline measures), immediately after, and later to follow-up.^24,25^ Such evaluation can also help to compare mobile quiz-games and other educational tools in terms of their effectiveness.

## Conclusion and implications

The huge success of Alipay’s quiz-game has demonstrated the great power and feasibility of smartphone games in engaging the public with food information. This innovative practice has converted consumers from passive information receivers to active participants, challenging the traditional one-way food risk communication practice with a more entertaining, interactive approach. The wide collaborations between digital platform operators/experts and food authorities should be encouraged. Further, the success of Alipay’s quiz-game and the crucial role of reach speak to the value of investing in advertising (if there is a budget for it), and forming partnerships with other organizations, influencers, and experts who can cross-promote the campaign at heavily subsidized or waived rates.

We live in a digital era where everyone’s opinion, whether correct or not, can be widely disseminated. As a means to proactively address and combat misinformation, it is critical that the food scientists and organizations speak the “digital dialect,” creating content that rivals the entertaining nature of its digital competitors, such as social media. Smartphone games are one such strategy that shows promise in its ability to reach and teach the public about food safety and nutrition. Given the extent of misinformation and ignorance among the public on food safety issues,^26^ it is imperative that other food scientists and organizations follow suit and create content that can compete in the digital space.
